# Coupling environment and physiology to predict effects of climate change on the taxonomic and functional diversity of fish assemblages in the Murray-Darling Basin, Australia

**DOI:** 10.1371/journal.pone.0225128

**Published:** 2019-11-27

**Authors:** Anielly Galego de Oliveira, Dayani Bailly, Fernanda A. S. Cassemiro, Edivando Vitor do Couto, Nick Bond, Dean Gilligan, Thiago F. Rangel, Angelo Antonio Agostinho, Mark J. Kennard

**Affiliations:** 1 Programa de Pós-Graduação em Ecologia de Ambientes Aquáticos Continentais, Núcleo de Pesquisas em Ictiologia, Limnologia e Aquicultura (NUPÉLIA), Universidade Estadual de Maringá, Maringá, PR, Brazil; 2 Programa de Pós-Graduação em Ecologia e Evolução, Universidade Federal de Goiás, Goiânia, GO, Brazil; 3 Universidade Tecnológica Federal do Paraná, Campo Mourão, PR, Brazil; 4 Centre for Freshwater Ecosystems, La Trobe University, Wodonga, Victoria, Australia; 5 NSW Department of Primary Industries–Fisheries, Batemans Bay Fisheries Office, Batemans Bay, New South Wales, Australia; 6 Australian Rivers Institute, Griffith University, Nathan, Brisbane, Queensland, Australia; Instituto Federal de Educacao Ciencia e Tecnologia Goiano - Campus Urutai, BRAZIL

## Abstract

This study uses species distribution modeling and physiological and functional traits to predict the impacts of climate change on native freshwater fish in the Murray-Darling Basin, Australia. We modelled future changes in taxonomic and functional diversity in 2050 and 2080 for two scenarios of carbon emissions, identifying areas of great interest for conservation. Climatic-environmental variables were used to model the range of 23 species of native fish under each scenario. The consensus model, followed by the physiological filter of lethal temperature was retained for interpretation. Our study predicts a severe negative impact of climate change on both taxonomic and functional components of ichthyofauna of the Murray-Darling Basin. There was a predicted marked contraction of species ranges under both scenarios. The predictions showed loss of climatically suitable areas, species and functional characters. There was a decrease in areas with high values of functional richness, dispersion and uniqueness. Some traits are predicted to be extirpated, especially in the most pessimistic scenario. The climatic refuges for fish fauna are predicted to be in the southern portion of the basin, in the upper Murray catchment. Incorporating future predictions about the distribution of ichthyofauna in conservation management planning will enhance resilience to climate change.

## Introduction

Climate change is considered one of the greatest threats to global biodiversity [1,2]. Impacts include rising air and water temperatures, sea level and greenhouse gases, major changes in regional rainfall and runoff patterns, and increases in the occurrence and severity of extremes events [3,4]. These shifts are expected to impact on the distribution, composition and phenology of species [1,5].

It is predicted that the biological impacts of climate change may be larger in freshwater systems than in terrestrial and marine systems, given their dendritic nature where aquatic organisms may not be able to reach cooler habitats or higher altitudes [6,7]. Faced with environmental-climatic alterations, obligate freshwater biota cannot move across landmasses or through oceans [5] and are restricted to aquatic habitats (river networks, floodplains wetlands, springs) [8]. Climate-driven changes in species distributions will have impacts on local community dynamics and diversity, including functional diversity (the biodiversity component related to ecological functions and services played by the species) [9], since the pattern in the distributions of functional traits (species morphological, structural and behavioural characteristics) are likely to change. Because functional traits can influence species performance (or fitness; [10]), the loss of functionally important species can modify ecosystem structure, function and resilience [11,12].

Fish are the most diverse group of freshwater vertebrates and play a central role in the structure and function of freshwater ecosystems [13]. They provide food for aquatic and terrestrial consumers (e.g. other fish, reptiles, mammals and birds), can regulate aquatic food webs, cycle nutrients and act as ecosystem engineers [14,15]. In addition, they are providers of valuable goods and services to humans [16]. However, the effects of climate change on the functional diversity of freshwater fish remain poorly understood, and the few studies that have used the functional characteristics of species to infer their sensitivity to climate change are mostly restricted to the northern hemisphere (see [9,17]).

Australia’s freshwater ichthyofauna is impoverished due to the country’s long isolation from other continents, combined with an arid climate and low rainfall, which result in widespread freshwater scarcity [18,19]. In Australia’s largest river basin, the Murray-Darling Basin, freshwater fishes are already exposed to numerous threats, such as flow regulation, habitat degradation, introduced species, exploitation and stocking [20–22] and may also be highly vulnerable to climate change. Native fish population sizes in the Murray-Darling Basin have been estimated to be 10% of their pre-European-settlement levels, and more than half of the Basin’s native fish species are now listed as threatened or of conservation concern [19]. The impacts of climate change on fish communities can be even greater for species-depauperate systems, in which the loss or the gain of a small number of species may lead to a disproportionate shift in assemblage composition [8]. Forecasting the potential impacts of climate change on fish species composition and functional diversity in the Murray-Darling Basin is crucial in identifying effective conservation strategies to preserve this already threatened ichthyofauna and the important ecosystem services they provide [9,23].

One way to anticipate the impacts of climate change on freshwater biodiversity within a functional context is to use correlative predictive models, usually referred to as species distribution models (SDMs) (or ecological niche models; see [24], followed by comparative analysis of the current and future functional structure of the assemblages. Although SDMs are useful for this goal, they typically disregard the influence of physiological traits of the species which can mediate spatial and temporal variation in their distributions in response to environmental change [25]. For example, thermal tolerance limits, especially the upper thermal limit, are essential to define species distribution changes in response to increasing temperatures associated with climate change. Incorporating such physiological information can provide a more mechanistic basis for predicting ecological responses to climate change [26].

Considering that climate is a fundamental factor determining the distribution of organisms at large spatial scales (reflecting the Grinnellian component of the ecological niche [27], and that tolerance limits determine whether the species are able to withstand the conditions imposed by an ever-changing environment, this study aimed to evaluate the effects of climate change on the distribution of native freshwater fish of the Murray-Darling Basin (MDB) using SDM’s combined with a physiological trait (the upper thermal tolerance limit). Firstly, we modelled the effects of future climate (2050 and 2080) on fish species richness, also investigating the responses of individual species in terms of whether they were predicted to undergo range expansion or contraction. Next, we analyzed the effects of climate change on functional diversity (functional richness, functional dispersion and functional uniqueness) of fish assemblages, identifying climatically suitable areas for species persistence as well as areas that preserve their existing functional character under future scenarios. Finally, we identified which functional trait characteristics may become dominant in fish assemblages in future, and those that may be lost as a result of climate change. By simultaneously considering taxonomic and functional attributes, and by coupling environment and physiology in estimation of species distribution, our study provides important information for guiding the spatial distribution of conservation efforts required in the Murray-Darling Basin in the light of potential effects of climate change on freshwater fish.

## Material and methods

### Study area

**Fig 1:**
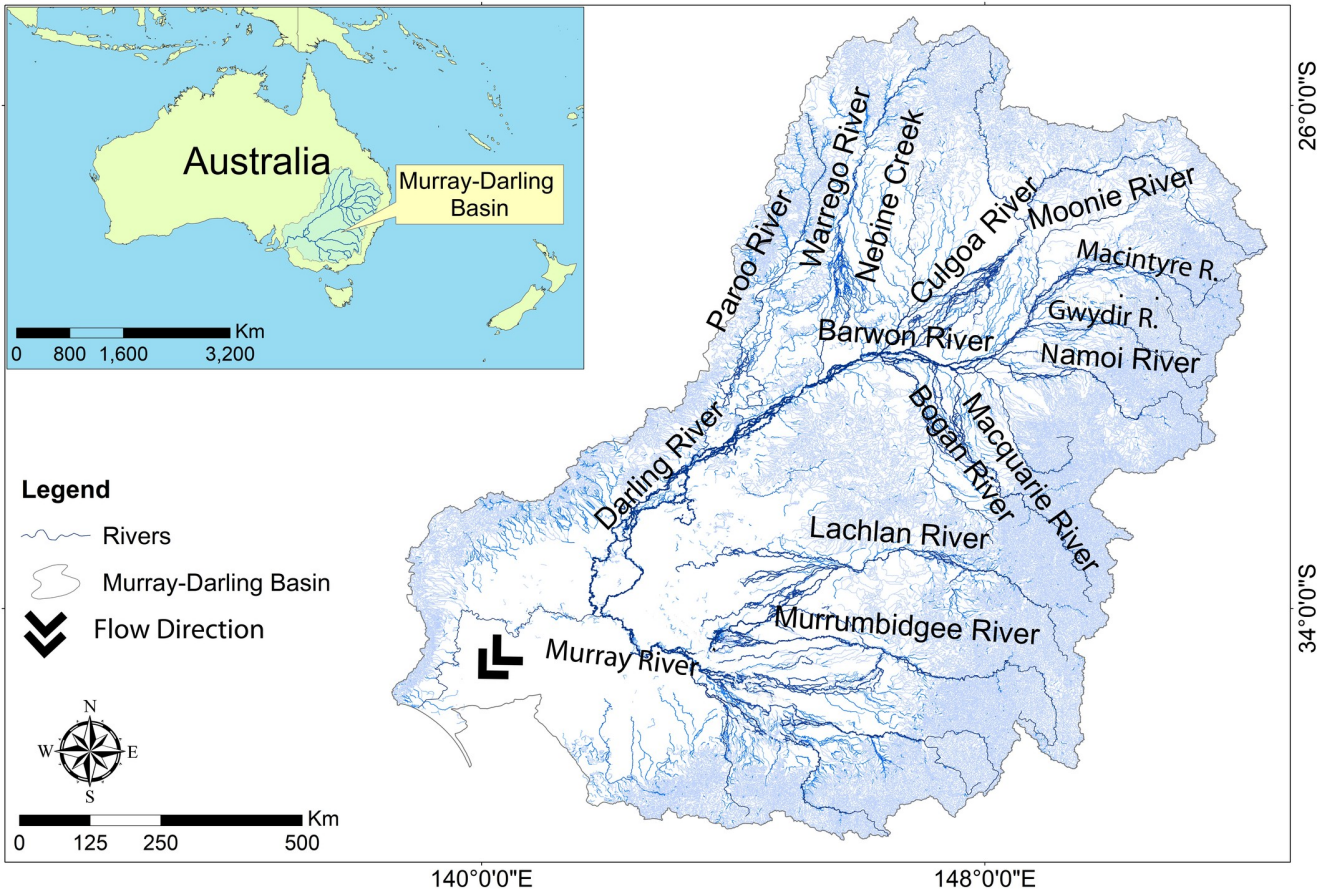
Map of Murray-Darling Basin showing the main rivers.

The Murray-Darling Basin (MDB) is a semi-arid basin in southeastern Australia (between the latitudes of 24 and 38°S) that covers more than a million square kilometers (1,063,000 km^2^), equivalent to 14% of Australia’s total area [19,28] (Fig 1). Despite its size, it has very low run-off and is one of the driest catchments in the world [28]. Because of the water storage and abstraction, only a third of natural mean annual discharge reaches the sea [15]. The MDB supports some of Australia’s most biologically and ecologically important floodplain and wetlands habitats, including 16 sites listed as Wetlands of International Importance under the Ramsar Convention [29]. Nearly two million people depend on the Basin’s resources and the value of its agricultural produce exceeds $24 billion each year [30].

### Species occurrence data

The fish fauna of MDB consists of 46 native freshwater fish species and 11 introduced species (not included in the analysis). Of the 46 native species, 16 are found only in the Murray-Darling Basin [19]. The analyses were performed with fish occurrence records only from within the MDB; these were obtained from state government agency fish distribution databases (Victorian Department of Sustainability and Environment and New South Wales Department of Fisheries) and sites surveyed as part of the Murray–Darling Basin Sustainable Rivers Audit [31]. We restricted our analysis to 4,347 sites surveyed between 1980 and 2010 for which reliable location and sampling information were available. Sampling involved a range of methods, including electrofishing and netting. In this study, we restricted analyses to those native species for which reliable information of functional traits and upper thermal tolerance limits are available (n = 23 species; see details below). Introduced species were excluded from all analyses. Occurrence records were mapped in a regular grid of 0.1° latitude and longitude (6,485 grid cells, each with approximately 11 km side and 120 km^2^ area) with a buffer of 10 km from each side of the river network. For each species, a binary matrix of presence (1) and pseudo-absence (0) was constructed from the occurrence data, which formed the biotic component for the modeling. Considering that previous studies have demonstrated increasing precision of predictive models with increasing prevalence (i.e. proportion of occupied survey points; [32], rare native species (i.e. those occupying < 4 grid cells) were excluded from all analyses. The final dataset for modelling included 23 species (Table A in S1 File) for which a total of 9,219 occurrence records were available (Fig A in S1 File); this resulted in 4,275 occupied grid cells.

### Predictor variables

We selected a set of ecologically relevant and minimally redundant environmental attributes as predictor variables in the SDMs (Table B in S1 File). The following bioclimatic predictors were used in the modeling process: annual mean temperature (TMEAN;°C), maximum temperature in the hottest month (TMAX;°C), minimum temperature in the coldest month (TMIN;°C), annual precipitation (PANN; mm), precipitation of wettest month (PMAX; mm) and precipitation of driest month (PMIN; mm). Temperature and precipitation have been chosen because they are the major climatic parameters determining the distribution of organisms on Earth [33]. As ectotherms, temperature is one of the most fundamental variables influencing the physiology of fishes, influencing physiological condition, development, growth rates, reproduction and behavior [34–36]. Precipitation determines aquatic habitat availability and the seasonal variations of droughts and floods, synchronizing biological events of species, such as migration, spawning, home range shifts and growth [36]. We also used the catchment topographic variable upstream flow path length (UFL) to represent variation in aquatic habitat availability [34,36] and altitude (ALT). The bioclimatic variables representing current conditions were obtained from WORLDCLIM (http://www.worldclim.org) and for future times from CCAFS (http://ccafs-climate.org; Research program on Climate Change, Agriculture and Food Security), both with a spatial resolution of 30 arc-seconds (~1 km). Catchment topography and altitude were obtained from a 9 second digital elevation model (DEM; [37]). All variables were averaged according to the grid of 0.1° resolution for obtaining the environmental layers. Absolute Pearson’s correlation coefficients among predictor variables did not exceed 0.7.

We have chosen two years, 2050 (mid of the century) and 2080 (end of the century) to predict the future scenarios of climate change over the fish distribution. TMEAN, TMAX, TMIN, PANN, PMAX and PMIN for future scenarios were extracted from the Intergovernmental Panel on Climate Change, Fifth Assessment Report (IPCC-AR5). Our predictions involved four Atmospheric-Ocean General Circulation Models (AOGCMs): CSIRO (Australia’s Commonwealth Scientific and Industrial Research Organization), MIROC (Model for Interdisciplinary Research on Climate), MRI (Meteorological Research Institute) and NCAR (National Center for Atmospheric Research) (see [38] for further details). The greenhouse gas concentration trajectory for each AOGCMs were based on the Representative Concentration Pathways (RCP), from a moderate—RCP 4.5 *stabilization* to a pessimistic scenario—RCP 8.5 *business-as-usual* of carbon emission. The difference between them consist in the assumptions they use about population, economic growth, energy consumption and sources and land use over this 21^st^ century [39]. The term ‘‘stabilization” here means an intermediate scenario of accumulation of greenhouse gases in the Earth’s atmosphere in future, rising until the mid of the century and after diminishing, and the term ‘‘pessimistic” means a scenario of high accumulation of greenhouse gases without efforts to contain the emissions [4]. When modeling species geographical distribution for future scenarios, we assumed temporal stationarity of UFL and ALT.

### Species distribution modeling

The matrices of species occurrences and climatic-environmental layers were used to calibrate multiple SDMs for each species, from which the environmental suitability and potential distribution of the species were modelled under current and future scenarios.

Given the conceptual and statistical particularities of different SDM approaches, a range of predictions can be generated, introducing uncertainty about which is the best model to represent the environmental suitability and the potential distribution of species [40]. To overcome this, we applied an ensemble forecasting approach to derive the consensus result amongst six SDM approaches (CONS, [41]). The main principle in using a consensus approach is that different sources of error affect each niche model in different ways, and the combination of its predictions tends to minimize errors and generate more robust predictions [40].

Our modeling protocol included six SDMs: BIOCLIM (BIOC; [42]), Euclidian Distance (EUCD; [43]), Gower Distance [43,44], Ecological Niche Factor Analysis (ENFA; [45]), Maximum Entropy (MAXE; [46]), and Genetic Algorithm for Rule-set Production (GARP; [47]). These models represent a wide variation in predictions due to the variety of statistical techniques. All six rely on the use of species presence-only and presence-background data. Only results of the consensus models (CONS) were retained for interpretation which means if a model had not a good performance according the evaluation and validation methods below described, it was not included in the final model.

The suitability matrices range from 0 to 1, in which values equal to 1 correspond to ideal habitat conditions and values equal to 0 correspond to suboptimal habitat conditions for the species [48]. For each SDM, the continuous suitability predictions were converted into binary vector (1/0) using a threshold that maximizes the sensitivity and specificity values in the Receiver Operating Characteristic curve (ROC curve; see Fielding and Bell 1997). The ROC curve is generated by plotting the fraction of true positives versus the fraction of false positives, at various threshold settings. The use of all possible thresholds avoids the need for a selection of a single threshold, which is often arbitrary. The area under the ROC curve (AUC) is often used as a single threshold-independent measure for model performance [49].

The species occurrence dataset was randomly divided into two subgroups: 75% of the data was used for calibration (training data) and 25% for validation/evaluation (test data). This procedure was performed 50 times to avoid biases in the calibration and evaluation data subgroups.

The distribution of each species in current climatic conditions was estimated using 300 predictions (6 SDMs x 50 randomizations). The simulations for future climatic conditions were estimated obtaining 1,200 predictions (6 SDM’s x 50 randomizations x 4 AOGCM’s) for each future scenario (2050 and 2080) and carbon emission scenario (RCPs 4.5 and 8.5), totaling 4,800 future predictions. This replication allowed us to generate a frequency of projections for each SDMs, which were weighted by the True Skill Statistics (TSS), i.e., better models according to this metric will have more weight in our consensus projections. The TSS statistic varies from -1 to +1, where values equal to +1 are a perfect prediction and values equal to or less than 0 are no better than random ones [49]. We used the majority consensus rule [40] to obtain the final consensus model for each species. This method considers the species present only in cells where at least 50% of the models retained in the ensemble predicted the species to be present. The modeling of species distribution was performed in the BioEnsembles computational platform [40].

Given that temperature tolerance limits are pivotal within the climate change context, we combined the correlative SDMs with independently derived estimates of the upper thermal tolerance of each fish species to further constrain species ranges estimates under each of the time (2050 and 2080) and carbon emission levels (RCPs 4.5 and 8.5). The combined model generated for each species provides an environmental envelope truncated by the upper thermal tolerance limit (i.e. the lethal maximum temperature), furnishing a potentially more reliable delineation of the geographic distribution of each species. This constrained delineation is likely to help avoid false predictions, particularly regarding potential future range shifts. Thus, for each species, the final range was constructed from the presence-absence matrices generated by CONS model, keeping presences only in cells whose maximum temperature is tolerable by the species (TMAX minor or equal to the lethal maximum temperature). As the bioclimatic variable used was air temperature, we used the model relating air temperature and water temperature proposed by [50] to estimate the latter in each grid cell. The lethal maximum temperature for each species was obtained from [51–53].

### Taxonomic diversity attributes and range size

To determine taxonomic diversity (species richness patterns), we employed the modeling strategy at the community level of “predict first, assemble later” (*sensu* [54]), in which the ranges of individual species are overlapped to obtain the number of species predicted in each cell.

The range size of each species was the sum of the number of occupied cells within the MDB. Temporal variations of the range size were analyzed by comparing the frequency distribution of the number of cells occupied by individual species. Taxonomic diversity and range size of individual species were obtained for current, 2050 and 2080 scenarios and for the different carbon emission levels, considering the outputs of our combined model (i.e. correlative models described by CONS truncated by the upper thermal tolerance limits).

### Functional diversity attributes

While the MDB has relatively few native freshwater fish species, those present represent a diversity of size, form and life history requirements [19,55]. We selected a set of morphological, behavioral, trophic and reproductive traits aimed at identifying the complementary functional aspects of the fish assemblage niche. The functional traits used were: (i) Maximum temperature in the warmest month within the range of species occurrence (TMAX), (ii) minimum temperature in the coldest month within the range of species occurrence (TMIN), maximum total body length (MAXL), (iii) vertical position in water column (benthic–VPBEN; non-benthic–VPNBEN), (iv) longevity (LONG), (v) age at maturation (AGEMAT), (vi) movement classification: non-movement–NON MOV; potamodromy–POTAMO (fishes that migrate between different sites in freshwater); amphidromy–AMPHID (fishes that regularly migrate between freshwater and the sea, in both directions, but not for the purpose to breeding); catadromy–CATAD, (freshwater fishes that migrate to the sea for the purpose of breeding), (vii) parental care (PARC), (viii) total fecundity (TFEC), (ix) egg size (EGGS), (x) trophic guild (herbivorous-detritivorous–HERB-DET; omnivorous–OMNI; invertivorous–INV; invertivorous-piscivorous–INV-PISC). A brief description of each functional trait can be found in Table C in S1 File. Trait assignments were based on a number of sources, including species accounts in comprehensive texts (i.e. [19,56–59]), species descriptions from the primary literature, state agency reports, university reports, graduate theses, and electronic databases available on the World Wide Web (e.g. FishBase). All trait information was assigned based on a majority of evidence rule, with preference given to adult female measurements where possible (see [60]) for more details on trait assignments).

From the presence and absence matrices derived from the consensus model, functional diversity was calculated for each time and carbon emission scenario. Functional diversity was obtained from three indices: Functional Richness (FRic–[61]), Functional Dispersion (FDis–[62]), the Functional Uniqueness (FUni–[63]). FRic represents the multidimensional volume occupied by the community and does not consider species’ abundances. FDis [64] is the mean distance weighted by abundance to the centroid (multivariate dispersion). For presence and absence data, where species have equal abundances, the Functional Dispersion is simply the mean distance to the centroid. Changes in FDis reflect changes in the species traits in relation to the center of functional space. FUni assumes that species with different traits perform distinct functions in the ecosystem. Thus, it is high when species have unique trait value combinations compared with each species of the pool (i.e. low redundancy). FUni (range 0 to 1) is the ratio between Rao’s entropy and the Simpson diversity index, relating observed functional diversity to the maximum value of dissimilarity of the community [63].

Indices were calculated in the R environment [65]. FRic and FDis were calculated using the function “dbFD” (distance based on functional diversity) from the FD package, proposed by [62]. The traits matrix had mixed variables (continuous and categorical–see Table C in S1 File), so we used Gower’s dissimilarity with Cailliez’s correction [66,67] for negative eigenvalues. FUni was calculated from the function “uniqueness” from [63]. All indices were calculated for each grid cell. The relationship between species richness and functional richness was evaluated through a Spearman correlation, since, according to the nature of this functional index, there is expected to be a high correlation between them [61].

From a T matrix (traits x grid coordinates; using SYNCSA R package, function “matrix.t”–[68], we performed the Indicator Value Analysis, which varies from 0 to 1 (IndVal; [69]), using carbon emission scenarios as a factor with five different levels). IndVal identifies which traits are significantly increased or decreased in frequency in future scenarios. Good indicator traits would be those that are both abundant in a specific scenario (specificity) and predominantly found in a scenario (fidelity). Because we only used presence and absence, we only used fidelity. The statistical significance level adopted was α = 0.05, and these analyses were performed in R environment, using the package labdsv, function “indval” [65].

## Results

### Taxonomic diversity attributes and range size

**Fig 2:**
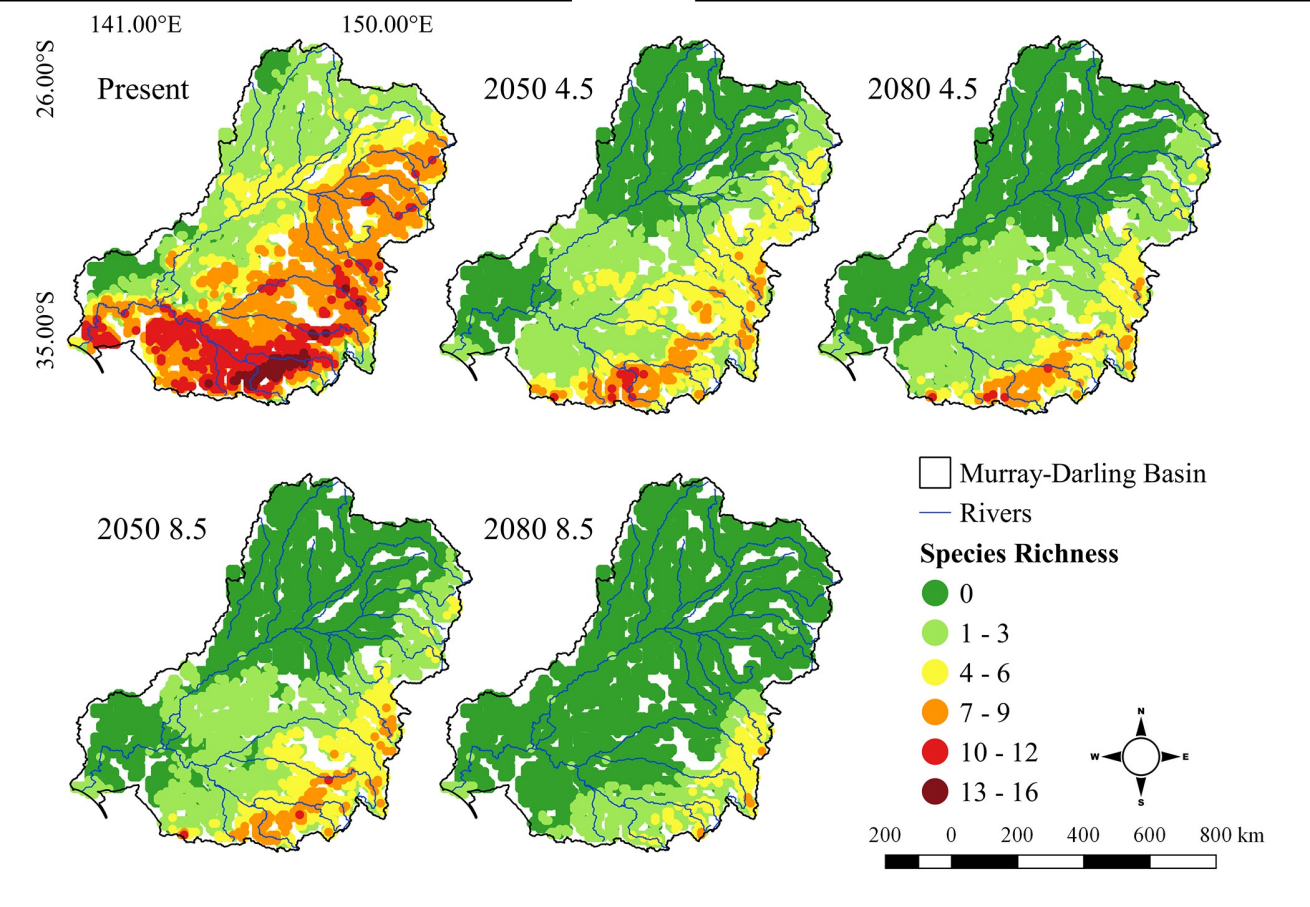
Species richness of fishes in Murray-Darling Basin in the present, 2050 and 2080 for RCPs 4.5 and 8.5.

Species richness predictions derived from the consensus model (CONS truncated by upper thermal tolerance limits of the species) identify the upper Murray River region as the richest in the basin (up to 16 species in a single cell) (Fig 2), emphasizing the high current climatic-environmental diversity within these drainages, and the suitability of this region for the ichthyofauna. Other catchments in the southeast of the basin (Lachlan and Murrumbidgee), southwest and northeast (e.g. Gwydir River catchment) have intermediate richness (up to 12 species). The rivers of the northwest presented the lowest ichthyofaunal richness (up to 6 species). However, future predictions produced by SDM’s and the physiological component revealed a marked loss of climatically suitable areas throughout much of the basin. The combined model suggests that the highest species richness will be restricted to a much reduced area in the southeastern upper Murray River and its major tributaries, with species losses intensifying toward the end of the century in the pessimistic scenario. In 2080 for RCP 8.5, the majority of richest cells tend to support only 5–6 fish species.

**Fig 3:**
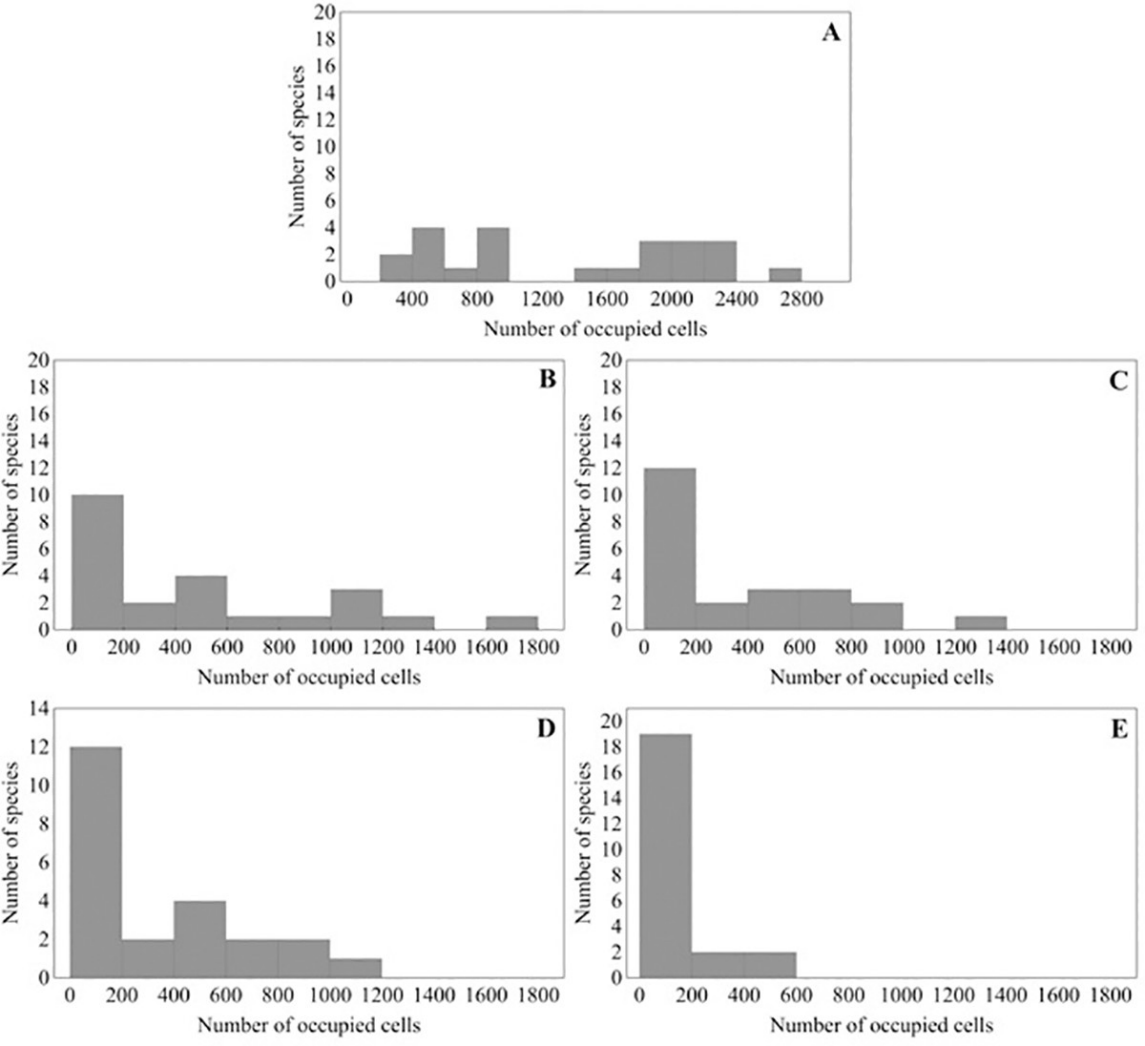
Current range size of fish species from Murray-Darling basin, Australia, and expected changes due to future climate changes (A = present, B = 2050 4.5, C = 2050 8.5, D = 2080 4.5, E = 2080 8.5).

Present-day fish species range sizes vary widely (200 to 2,800 occupied cells) in the MDB (Fig 3A). Future predictions indicate a marked range contraction under climate change for all species (Fig 3B, 3C, 3D and 3E). In the most pessimistic scenario in 2080, only four species (*Hypseleotris klunzigeri*, *Macquaria ambigua*, *Ambassis agassizii* and *Tandanus tandanus*), occupied more than 200 grid cells (Fig 3E). For *Galaxias maculatus* the range loss reached 100% in 2050 (RCP 8.5), with five species showing similar declines by 2080 (RCP 8.5) (*Galaxias maculatus*, *Galaxias oliros*, *Maccullochella macquariensis*, *Neosilurus hyrtlii*, *Philypnodon grandiceps*). Similarly, *Bidyanus bidyanus* and *Philypnodon macrostomus* were constrained to a single cell. These range retractions represent a loss of at least 25% of the species analysed here and 13% of the total of native fish species present in MDB. Not one species had its distribution extended in the future scenarios.

### Functional diversity

**Fig 4:**
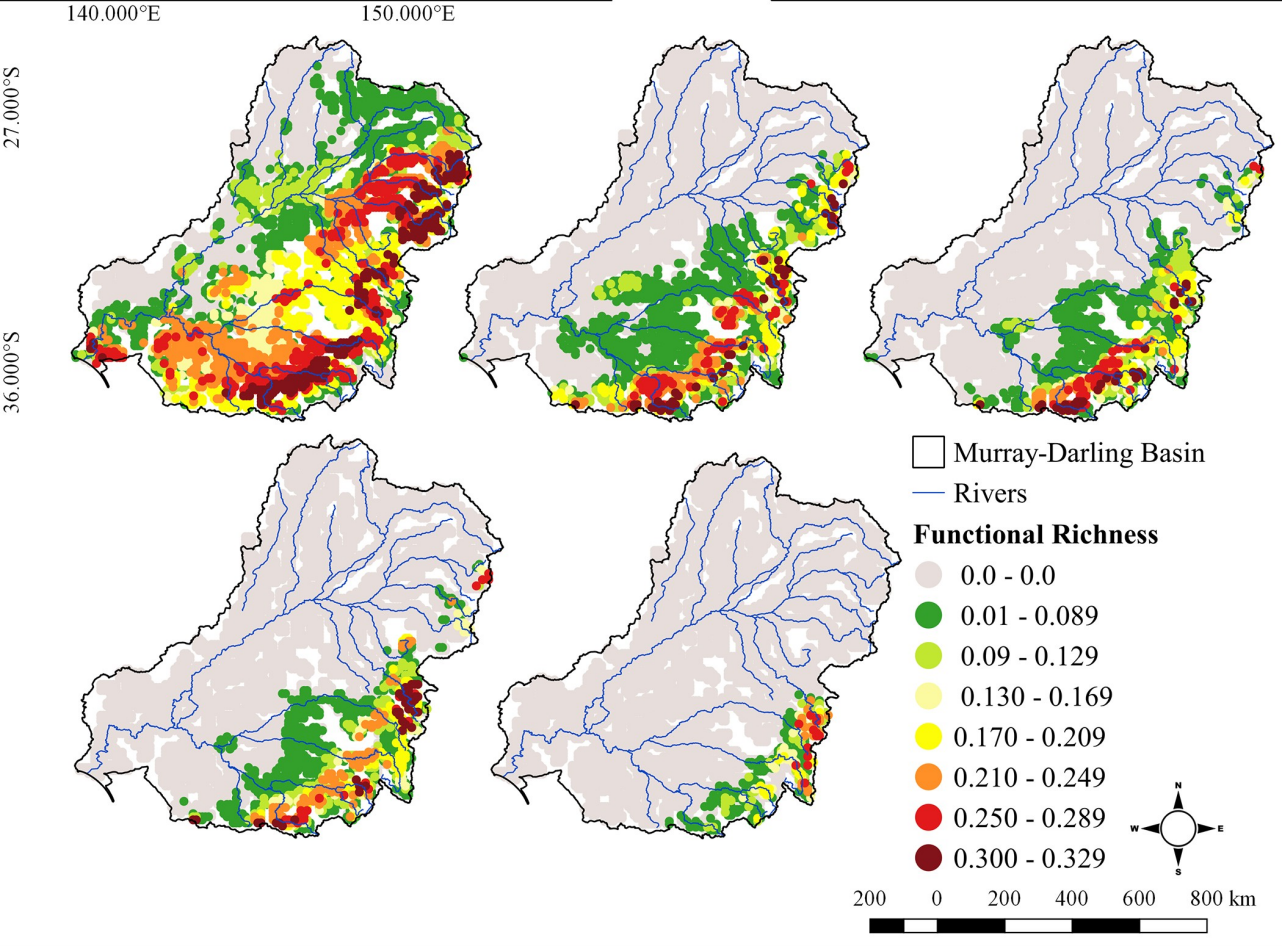
Species functional richness (FRic) of fishes in Murray-Darling Basin in the present, 2050 and 2080 and different RCPs (4.5 and 8.5) scenarios.

**Fig 5:**
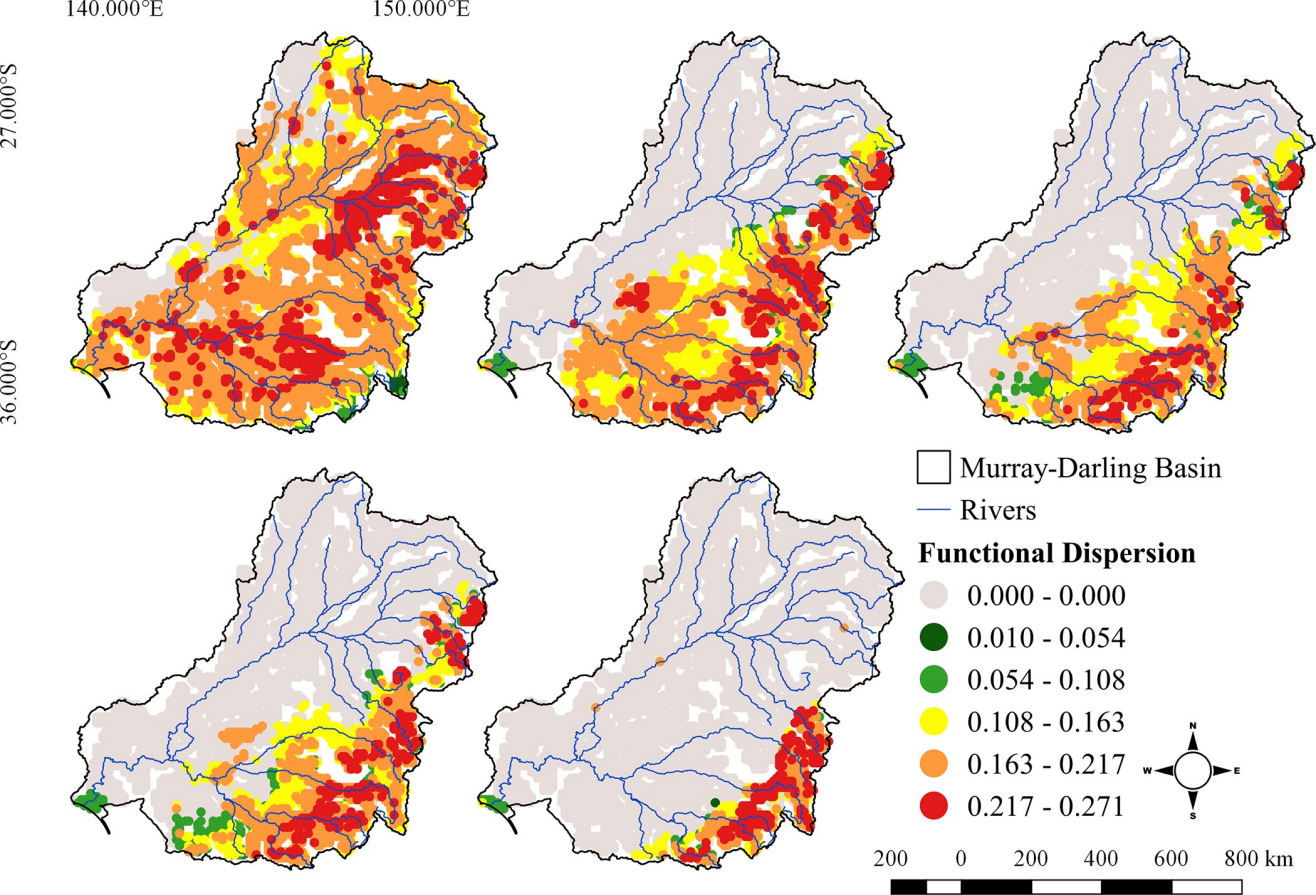
Functional dispersion (FDis) of fishes in Murray-Darling Basin, in the present, 2050 and 2080 and different RCPs (4.5 and 8.5) scenario.

Taxonomic and functional richness were positively correlated for all times and scenarios (r > 0.81, p < 0.05 for all pairwise comparisons), indicating that cells with higher taxonomic richness usually also had higher functional richness. The highest values of functional richness calculated from the combined model, were found in the northeastern part of Murray-Darling Basin; in the Macintyre, Gwydir and Namoi Rivers despite these regions only having intermediate levels of Species Richness, as well in the southeastern part of the MDB, in the upper reaches of the Murray and Murrumbidgee Rivers (Fig 4). There was a marked reduction in the number of cells with highest values of FRic (~ 0.30–0.33; Fig 5) in future scenarios, with the greatest values of FRic restricted to a few cells in the southeastern part of the basin, especially under the most pessimistic scenario (RCP 8.5).

The highest values of FDis corresponded with the highest values of FRic in the current scenario. Rivers from the Northeast region (e.g. Macintyre, Gwydir, Namoi) showed the highest FDis values. The upper Murray River and its major tributaries such as the Ovens and Goulburn Rivers also exhibited high functional dispersion values, and in the future, the trend of constraining of high values toward high altitudes in the Southeast region was the same for taxonomic and functional indices (Fig 5).

**Fig 6:**
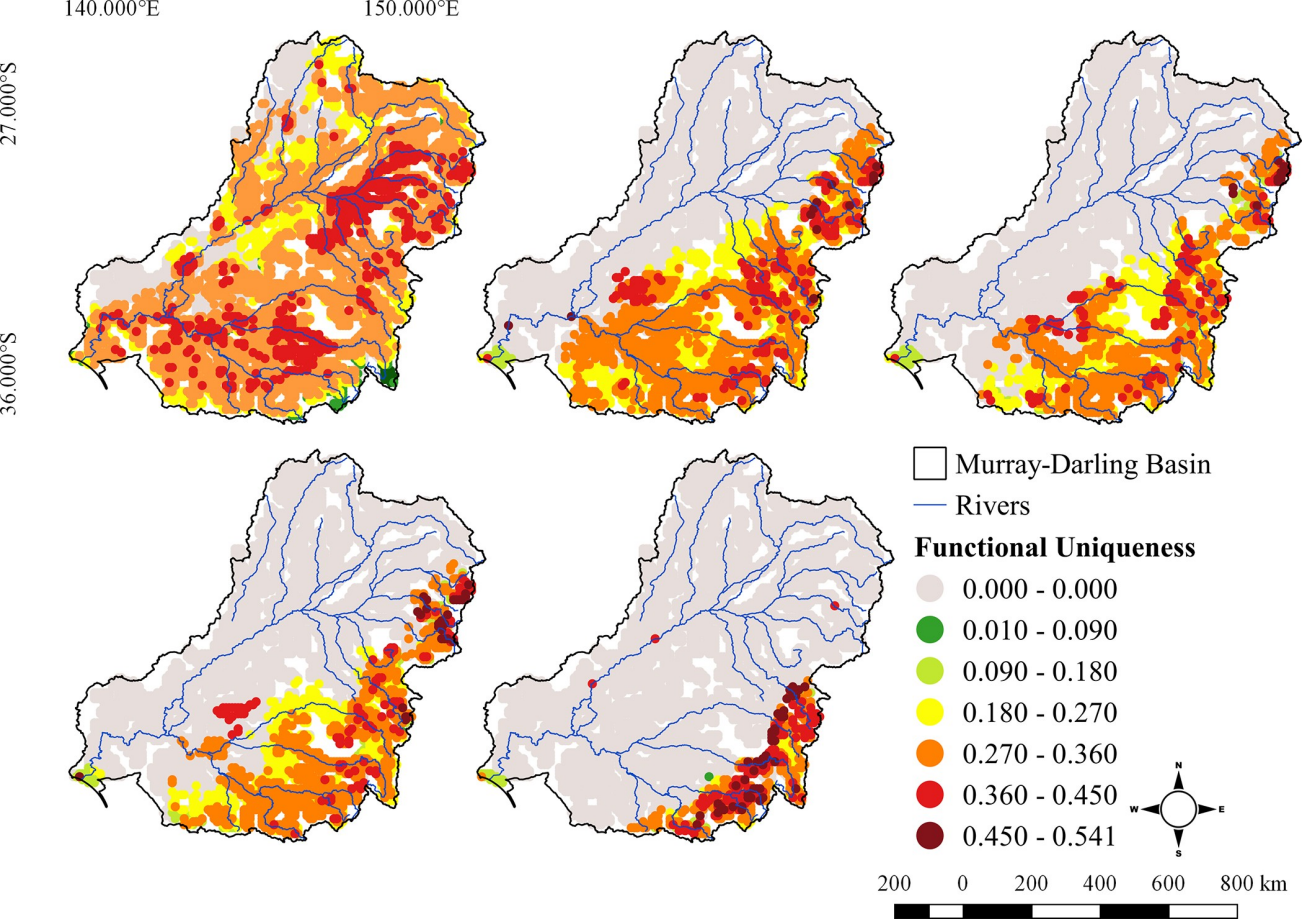
Functional uniqueness (FUni) of fishes in Murray-Darling Basin in the present, 2050 and 2080 and different RCPs (4.5 and 8.5) scenarios.

Regions of the MDB with highest values of FUni for the present scenario were the Condamine River catchment upstream of St George, the lowlands reaches of Northeast Region catchments, the lower Darling River together with small areas of some of the eastern rivers (e.g the Macquarie-Castlereagh; Fig 6). This spatial pattern differs from both species richness and functional richness for the current scenario. These areas (North and Northeast) are also predicted to be the most heavily affected in the future. For 2050, areas of high FUni contract the very upper reaches only of smaller rivers such as the Border Rivers, Gwydir and Namoi are predicted to retain the highest functional uniqueness, while for 2080 the highest values were predicted in the upper reaches of the Macquarie, Lachan, Murrumbidgee and Murray rivers, following the taxonomic and functional richness tendencies (Fig 6).

### Indicator value analysis

**Fig 7:**
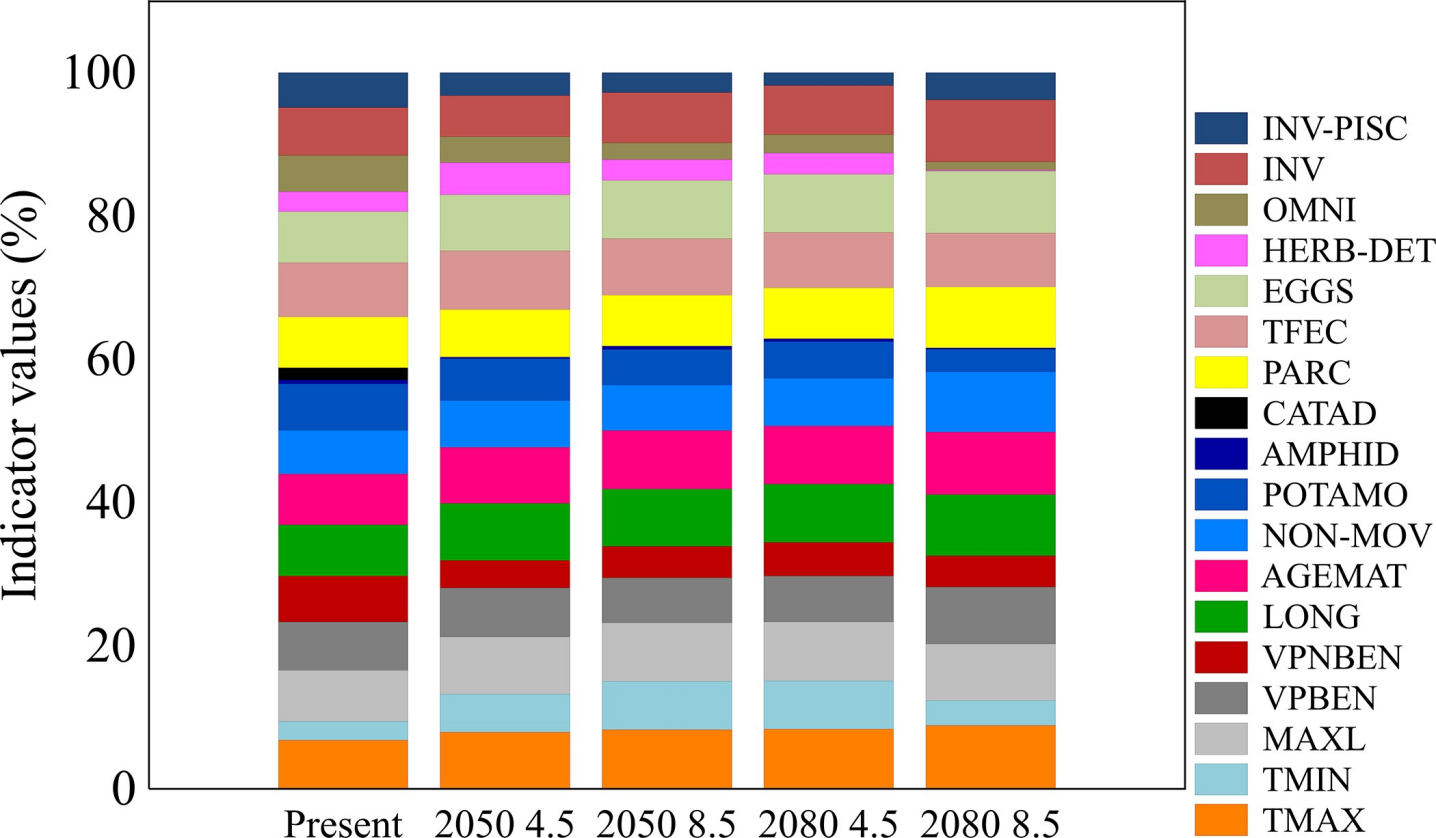
Indicator values indicating the proportion (0 to 100%) of contribution of each trait for fish species in different years (2050 and 2080) and RCP’s (4.5 and 8.5) for the Murray-Darling Basin, Australia. Higher values indicate traits predominantly found in this scenario (i. e. fidelity). (TMAX = Maximum temperature; TMIN = Minimum temperature; MAXL = Maximum total length; VPBEN = Vertical position benthic; VPNBEN = Vertical position non-Benthic; LONG = Longevity; AGEMAT = Age of maturation; NON-MOV = Non-movement; POTAMO = Potamodromous; AMPHID = Amphidromous; CATAD = Catadromous; PARC = Parental are; TFEC = Total fecundity; EGGS = Number of eggs; HERB-DET = Herbivorous-detritivorous; OMNI = Omnivorous; INV = Invertivorous; INV-PISC = Invertivorous-piscivorous).

All functional traits had their value of contribution significantly diminished in the future scenarios (p < 0.5) relative to present condition, as the results of IndVal showed (Table D in S1 File). Catadromy was the most affected trait with no relevant contribution in any future time and carbon emission scenario, followed by amphidromy, vertical position non-benthic, herbivorous-detritivorous and omnivorous. Nonetheless, in relation to the contribution of each trait composing the future scenarios in proportion (%—Fig 7) it was possible to note a subtle rising of maximum temperature, minimum temperature, eggs, total fecundity, vertical position benthic, longevity, non-movement, parental care, invertivorous, invertivorous-piscivorous at least in the contribution of composing the pessimistic scenario in 2080.

## Discussion

Species distribution models are widely used to forecast the impact of climate change on species distribution, including freshwater organisms [7,9,36]. Despite several advantages (see [48,70]), SDMs present limitations, especially those related to predictive uncertainties as aforementioned. In addition, the correlative approach of SDMs is exclusively focused on environmental-species occurrence correlations, ignoring physiological characteristics of species, which are traditionally addressed by mechanistic modeling (see [71,72]. In this sense, studies suggest the predictive outcomes should be substantially improved if the models take into account mechanistic elements, rather than using correlation alone [73,74].

Our study predicts a severe negative impact of future climate change over both taxonomic (species richness) and functional components of the ichthyofauna of the Murray-Darling Basin. Although this study focused on 23 of the 46 native species occurring in the basin, the general pattern of response to climate change we find is likely to be same for the other species of fish. Species distribution modelling combined with a physiological parameter and followed by a functional analysis, suggest a loss of climatically suitable areas, leading to less cells with the highest values of species richness, the contraction of range and loss of function of the majority of freshwater fishes of the MDB. These findings imply that under climate change, local extinction rates can increase considerably throughout the basin. Impacts are predicted to be particularly high along the north and northeaster regions, where high rates of loss of climatically suitable areas are projected by the end of the century. The upper catchments of the southern Basin, especially the upper Murray, Mitta Mitta, Goulburn and Campaspe Rivers stand out as potential climate refuges in the future scenarios, showing the highest values of retained species richness and functional diversity.

The southern portion of the MDB basin (Murray River catchment) currently supports a larger number of fish species than the arid western and northern parts of the basin (Darling River catchment), where rivers can dry to isolated waterholes in low-rainfall years [19]. Although the southeast portion of Murray-Darling Basin is predicted to retain the highest number of species and greatest functional diversity in the face of predicted climate change, our results also predict great losses of habitat suitability, with regions of greater richness retracting to higher altitudes as climate refuge. Indeed, favorable climatic-environmental conditions at higher altitudes in the face of climate change have been documented in several studies ([75–77]). For temperate species of stream fish in France, predictions suggest systematic species range shifts towards higher elevations and upstream reaches basin in response to climate change, with mean shifts in range center of 13.7 m decade^-1^ and 0.6 km decade^-1^, respectively [78]. It is important note that the displacement of species-friendly conditions encounters land limitations once the continental area does not expand as well as the mountaintops [79].

Despite species distributions are generally predicted to shift towards higher latitudes and altitudes as a result of climate changed induced shifts of bioclimatic variables [77,80] some components of assemblages can move in the opposite direction, or simply do not exhibit a retraction of their ranges [81]. These species-specific responses could uncouple important species interactions, such as the regulation of population abundance, nutrient cycling and habitat creation, affecting ecosystem processes and function [82].

Range contraction of fish assemblages was predicted in all future climate scenarios. Declines in species distribution occur because species have particular environmental requirements and physiological characteristics, directly influencing their adaptive responses to the environment [83]. For freshwater fish, these attributes are usually habitat conditions, especially temperature, dissolved oxygen and hydrology [84–86]. Habitats become unsuitable when conditions vary beyond tolerable limits of the species. When this occurs, metabolism and individual performance of organisms (especially ectotherms) are affected, decreasing the intrinsic rate of population growth and leading to extinction scenarios over time [36]. Under our simulations, the most alarming scenario is that five fish species are predicted to lose 100% of environmental suitable habitat within the MBD by the end of century. Two of these; *Galaxias oliros* (obscure galaxias) and *Maccullochella macquariensis* (trout cod), represent global extinctions given that both species are endemic to the MDB [19]. Similarly critical is the status of *Bidyanus bidyanus* (silver perch) which is considered as vulnerable species by International Union for Conservation of Nature (VU-IUCN), and listed as critically endangered under federal legislation in Australia.

Even though the decrease of climatic-environmental suitability and range size does not necessarily imply extinction of a species (see below the importance of physiologic plasticity), it is consensual in conservation biology that a decrease in the extent of occurrence or area of occupancy reflects increasing extinction risk [87]. In the face of predicted climate change, the persistence of species in their original ranges will be dependent on the degree of genetic diversity, physiological and phenotypic plasticity [88]. In this sense, historic-evolutionary hypothesis explaining diversity gradients postulates that due to the climatic stability of lower latitudes, tropical species evolve to have narrow thermal tolerances and that due to the climatic instability of high latitudes, temperate species evolve to have broader thermal tolerances [89,90]. As a consequence, tropical species are likely to be more vulnerable to changes in global climate due their narrow thermal niches [91,92]. Because the MDB is a temperate basin, it would be expected high tolerance of fish species to global warming forces. Nonetheless, we found that for many MDB fish species, the upper lethal temperature was lower than future temperature predictions. The fragility of temperate fish facing climate alteration was also pointed out by [11]. Evaluating plasticity to upper tolerance temperature of fishes, these authors found that species occupying higher latitudes showed no greater acclimatization capacity than those living at lower latitudes, interposing to historic-evolutionary hypothesis.

Regions of the MDB showing the highest current values for functional dispersion and uniqueness (e.g. the lower Darling River and the upper Condamine River catchment) also have the lowest fish species richness. In this circumstance, it is more likely that the loss of a given species will have large impacts on ecosystem processes and functionality. In this context, the conservation of these areas is arguably as important as protecting the richest areas. The future scenarios of *stabilization* (RCP 4.5) predicted high functional uniqueness in some smaller rivers in the northeast region (e.g. Gwydir and Lachan Rivers), however, in the *business-as-usual* scenarios (RCP 8.5) it was primarily the upper Murray River and Murrumbidgee Rivers in the southeastern region of the MDB that displayed more distinct assemblages. Such areas will become increasingly significant for conservation, because high values of functional uniqueness contribute disproportionately to maintaining a high level of functional diversity than species having common traits shared by other species (i.e. high functional redundancy) and may actually help to stabilize ecosystem processes as a result of functional niche complementarity [17]. Many studies have reported reductions in functional diversity of assemblages in response to anthropogenic impacts, driven by replacement of specialist taxa by more generalist species, resulting in functional homogenization [93,94].

The reduction of all functional traits under future climate scenarios tends to imply declines in the ability of fish assemblages to continue to provide comparable ecosystem services. Consistent with previous studies evaluating climate change responses of ichthyofauna in the MDB [20,52], some functional traits even decreasing in response to the loss of some species, can enlarge the proportional contribution within the future scenarios, as we observed in our most pessimistic (2080 8.5). When considered only future scenarios, the traits AGEMAT, EGG, TMAX, TMIN, LONG, NON-MOV, PARC, INV, INV-PISC and VPBEN tend to show better performance, especially in the pessimistic *business as usual* carbon emission scenario at the end of the century.

Although we did not measure ecological processes linked to ecosystem functioning directly, we can make some predictions. Loss of, or a decline of fish taxa with large body length, as suggested here, may compromise biomass and affect recreational fisheries that generate substantial socio-economic benefits. The larger fish species of the MDB, such as the Murray cod, golden perch, silver perch and freshwater catfish have historically provided an important food source for Aboriginal people and European settlers [95], supplying recreational activities and the commercial fish industry. In addition, the potential extirpation of species displaying traits such as catadromy, amphidromy, potamodromy, herbivory-detritivory and omnivory in 2080 and the severe reduction in vertical position non-benthic, and species tolerants to low temperatures (TMIN) under the pessimistic emission scenario warns of potential degradation of ecosystem functioning. The loss of species traits related to movement between distinct habitats implies a reduction in the transfer of energy, biomass and nutrients [96]. Despite not being entirely lost from the MDB based on our models, potamodromous fish also suffer a significant decline. The life-cycles of many potamodromous fishes are flow-dependent [97]. Forecasts of future declines in rainfall and runoff in MDB [98,99], suggest flow events triggering large-scale movements will decline. The loss of the traits herbivorous-detritivorous and omnivorous can alter the trophic structure of stream food webs, leading to cascades that have not been captured by predictions arising from SDMs [100].

The southeast region, which represents the main climate refuge of the MDB fish diversity is already affected by several contemporary impacts. Anthropogenic alterations of flow (regulation and consumptive use), fragmentation (by dams and weirs) and land use intensification (causing pollution and sedimentation) are significant in the southern MDB, but are not restricted to this area alone [19]. Another challenge to conservation of native fish in the MDB under climate change relates to the high number of introduced fish species in the basin. Although some studies have revealed diminishing impacts of invasive species under climate change [36,101], other indicate that future climate may exacerbate the threat posed by invasive species [102]. In Australia, introduced stream fish of Victorian streams were predicted to experience both contraction (*Salmo trutta* and *Oncorhynchus mykiss*) and expansion (*Gambusia holbrooki* and *Misgurnus anguillicaudatus*) of their ranges [21]. The MDB shows 11 invasive species (23% of the total), thus it is possible that the functional characteristics of these species may be favorably selected in the future. It is possible that introduced species may increase their dominance in climatic refuges intensifying biotic interactions such as predation and competition, with native species. The difficulty of predicting how interactions among species will be altered under future climate scenarios are reported in several papers (e.g. [103]). Thus, further research is needed to assess to what extent invasive species of MDB can spread their ranges considering future environmental conditions.

In conclusion, this paper models the response of taxonomic and functional diversity of freshwater fishes of the Murray-Darling Basin, Australia, under two carbon emission scenarios (*stabilization* and *business-as-usual*) for the years 2050 and 2080. The addition of physiological parameters representing the upper thermal tolerance limit of individual fish species offers some advantages over correlative modelling alone, because it can indicate locations in geographic space with a heat safety margin for species survival, thus guiding conservation efforts to the most promising areas. Our study suggests that native fish assemblages of the Murray-Darling Basin are sensitive to climate change, given the pronounced range contractions, species extinctions and changes to ecosystem functionality predicted to occur between now and the end of the century. Currently, there is a considerable effort being made to rehabilitate and sustainably manage the Basin. Initiatives such as the Native Fish Strategy for the Murray-Darling Basin (2003–2013) and Murray-Darling Basin Plan (2012 and ongoing), have outlined holistic targets for rehabilitating native fish including through investment in the provision of environmental flows, the installation of fishways, physical habitat restoration, the management of non-native fish species, and fish translocations and stocking [55]. From our predictions, we emphasize the critical need to begin to incorporate potential range shifts when undertaking such investments.

## Supporting information

S1 File **Table A. Fish species of the Murray-Darling Basin considered in this study.** The list is composed of fish species occupying at least four grid cells and for which functional traits and upper thermal tolerance limit (lethal maximum temperature) data are available.
**Table B. Environmental attributes used as predictor variables in the species distribution models**.
**Table C. Brief description of the traits used in the functional analysis**.
**Fig A. Species occurrence data in the Murray-Darling Basin, Australia**.
**Table D. Indicator values for species’ functional traits in the different scenarios.** Higher values represent a high proportional trait representation (i.e. fidelity) in a given scenario.
(DOCX)
