# Correction: Coupling environment and physiology to predict effects of climate change on the taxonomic and functional diversity of fish assemblages in the Murray-Darling Basin, Australia

**DOI:** 10.1371/journal.pone.0229679

**Published:** 2020-02-20

**Authors:** The PLOS ONE Staff

The first author's initials appear incorrectly in the citation. The publisher apologies for the error. The correct citation is: Oliveira AGd, Bailly D, Cassemiro FAS, Couto EVd, Bond N, Gilligan D, et al. (2019) Coupling environment and physiology to predict effects of climate change on the taxonomic and functional diversity of fish assemblages in the Murray-Darling Basin, Australia. PLoS ONE 14(11): e0225128. https://doi.org/10.1371/journal.pone.0225128.

## References

[1] Galego de Oliveira A, Bailly D, Cassemiro FAS, Couto EVd, Bond N, Gilligan D, et al (2019) Coupling environment and physiology to predict effects of climate change on the taxonomic and functional diversity of fish assemblages in the Murray-Darling Basin, Australia. PLoS ONE 14(11): e0225128 10.1371/journal.pone.0225128 31774852 PMC6880973

