# Supplementary material for: Coupling environment and physiology to predict effects of climate change on the taxonomic and functional diversity of fish assemblages in the Murray-Darling Basin, Australia
Source: PLoS One. 2019 Nov 27;14(11):e0225128. doi: 10.1371/journal.pone.0225128 (PMC6880973; doi:10.1371/journal.pone.0225128)
Supplement: S1 File — Table A. Fish species of the Murray-Darling Basin considered in this study. The list is composed of fish species occupying at least four grid cells and for which functional traits and upper thermal tolerance limit (lethal maximum temperature) data are available. Table B. Environmental attributes used as predictor variables in the species distribution models. Table C. Brief description of the traits used in the functional analysis. Fig A. Species occurrence data in the Murray-Darling Basin, Australia. Table D. Indicator values for species’ functional traits in the different scenarios. Higher values represent a high proportional trait representation (i.e. fidelity) in a given scenario. (DOCX) [file pone.0225128.s001.docx]

**Coupling environment and physiology to predict effects of climate change on the taxonomic and functional diversity of fish assemblages in the Murray-Darling Basin, Australia**

Anielly Galego de Oliveira¹*, Dayani Bailly¹, Fernanda A. S. Cassemiro², Edivando Vitor do Couto³, Nick Bond^4^, Dean Gilligan^5^, Thiago F. Rangel², Angelo Antonio Agostinho¹, Mark J. Kennard^6^

¹Programa de Pós-Graduação em Ecologia de Ambientes Aquáticos Continentais, Núcleo de Pesquisas em Ictiologia, Limnologia e Aquicultura (NUPÉLIA), Universidade Estadual de Maringá, PR, Brazil

² Programa de Pós-Graduação em Ecologia e Evolução Universidade Federal de Goiás, Goiânia, GO, Brazil

³Universidade Tecnológica Federal do Paraná, Campo Mourão, PR, Brazil

^4^Centre for Freshwater Ecosystems, La Trobe University, Wodonga, Victoria 3690, Australia

^5^ NSW Department of Primary Industries – Fisheries, Batemans Bay Fisheries Office, Batemans Bay, New South Wales 2536, Australia.

^6^Australian Rivers Institute, Griffith University, Nathan, Queensland 4111, Australia

*Corresponding author:

Anielly G. Oliveira; e-mail: anielly_oliveira@hotmail.com

Telephone number: +55 44 30114607

Table A. Fish species of the Murray-Darling Basin considered in this study. The list is composed of fish species occupying at least four grid cells and for which functional traits and upper thermal tolerance limit (lethal maximum temperature) data are available.

| Order | Family | Species | Common name | Number of occupied cells and relative frequency of occurrence | Conservation Status (IUCN*) |
| --- | --- | --- | --- | --- | --- |
| Atheriniformes | Atherinidae | *Craterocephalus stercusmuscarum*  (Günther 1867) | Un-specked hardyhead | 394 (6.07%) |  |
|  | Melanotaenidae | *Melanotaenia fluviatilis*  (Castelnau 1878) | Murray-Darling rainbowfish | 526 (8.11%) |  |
| Perciformes | Chandidae | *Ambassis agassizii*  Steindachner 1867 | Olive perchlet | 32 (0.49%) | DD |
|  | Clupeidae | *Nematalosa erebi*  (Günther 1868) | Bony herring | 860 (13.26%) | LC |
|  | Eleotridae | *Hypseleotris klunzingeri*  (Ogilby 1898) | Western carp gudgeon | 1291 (19.90%) |  |
|  |  | *Mogurnda adspersa*  (Castelnau 1878) | Southern purple-spotted gudgeon | 34 (0.52%) |  |
|  |  | *Philypnodon grandiceps*  (Krefft 1864) | Flat-headed gudgeon | 484 (7.46%) |  |
|  |  | *Philypnodon macrostomus*  Hoese and Reader 2006 | Dwarf flat-headed gudgeon | 16 (0.24%) |  |
|  | Percichthyidae | *Gadopsis marmoratus*  Richardson 1848 | River blackfish | 459 (7.07%) |  |
|  | Percichthydae | *Maccullochella macquariensis*  (Cuvier 1829) | Trout cod | 141 (2.17%) | EN |
|  |  | *Maccullochella peelii*  (Mitchell 1838) | Murray cod | 731 (11.27%) | CR |
|  |  | *Macquaria ambigua*  (Richardson 1845) | Golden perch | 1071 (16.51%) |  |
|  |  | *Macquaria australasica*  Cuvier 1830 | Macquarie perch | 110 (1.69%) | DD |
|  |  | *Nannoperca australis*  Günther 1861 | Southern pygmy perch | 167 (2.57) |  |
|  | Terapontidae | *Bidyanus bidyanus*  (Mitchell 1838) | Silver perch | 251 (3.87%) | VU |
|  |  | *Leiopotherapon unicolor*  (Günther 1859) | Spangled perch | 469 (7.23%) |  |
| Salmoniformes | Galaxiidae | *Galaxias maculatus*  (Jenyns 1842) | Common galaxias | 34 (0.52%) | LC |
|  |  | *Galaxias olidus*  Günther 1866 | Mountain galaxias | 642 (9.88%) |  |
|  |  | *Galaxias oliros*  Raadik 2014 | Obscure galaxias | 61 (0.94%) |  |
|  |  | *Galaxias arcanus*  Raadik 2014 | Riffle galaxias | 32 (0.49%) |  |
|  | Retropinnidae | *Retropinna semoni*  (Weber 1895) | Australian smelt | 1043 (16.08%) |  |
| Siluriformes | Plotosidae | *Neosilurus hyrtlii*  Steindachner 1867 | Hyrtl’s tandan | 59 (0.90%) |  |
|  | Plotosidae | *Tandanus tandanus*  (Mitchell 1838) | Freshwater catfish | 265 (4.08%) |  |

*IUCN = Internation Union for Conservation of Nature

Wager, R. 1996. *Maccullochella peelii*. *The IUCN Red List of Threatened Species* 1996: e.T12576A3361423. <http://dx.doi.org/10.2305/IUCN.UK.1996.RLTS.T12576A3361423.en>.

Wager, R. 1996. *Macquaria australasica*. *The IUCN Red List of Threatened Species* 1996: e.T12581A3361826. <http://dx.doi.org/10.2305/IUCN.UK.1996.RLTS.T12581A3361826.en>.

Wager, R. 1996. *Bidyanus bidyanus*. *The IUCN Red List of Threatened Species* 1996: e.T2804A9482704. <http://dx.doi.org/10.2305/IUCN.UK.1996.RLTS.T2804A9482704.en>.

David, B., West, D, Franklin, P., Allibone, R, Ling, N., Hitchmough, R. & Crow, S. 2014. *Galaxias maculatus*. *The IUCN Red List of Threatened Species* 2014: e.T197279A2481214. <http://dx.doi.org/10.2305/IUCN.UK.2014-3.RLTS.T197279A2481214.en>.

Table B. Environmental attributes used as predictor variables in the species distribution models

| Predictor variables (Acronyms) | Predictor variables | Data source  Present/Future |
| --- | --- | --- |
| TMEAN; °C | Annual mean temperature | Worldclim/CCAFS |
| TMAX; °C | Maximum temperature in the hottest month | Worldclim/CCAFS |
| TMIN; °C | Minimum temperature in the coldest month | Worldclim/CCAFS |
| PANN; mm | Annual precipitation | Worldclim/CCAFS |
| PMAX; mm | Precipitation of the wettest month | Worldclim/CCAFS |
| PMIN; mm | Precipitation of driest month | Worldclim/CCAFS |
| UFL | Upstream Flow Path Length | Stein et al., 2014 |
| ALT (m) | Altitude | Stein et al., 2014 |

Data source:

<http://www.worldclim.org>

<http://ccafs-climate.org>

Stein, J.L., Hutchinson, M.F., Stein, J.A., 2014. A new stream and nested catchment framework for Australia. Hydrol. Earth Syst. Sci. 18, 1917–1933.

Table C. Brief description of the traits used in the functional analysis.

| **Biological Attribute** | Description | Code |
| --- | --- | --- |
| ***Body morphology*** |  |  |
| Maximum length | Maximum total body length (cm) | MaxL |
| **Behaviour** |  |  |
| Vertical position | Benthic | VPBEN |
|  | Non-benthic | VPNBEN |
| **Life history** |  |  |
| Longevity | Maximum potential life span (years) (Females) | LONG |
| Age at maturation | Mean age at maturation (years) (Females) | AGEMAT |
| Parental care | Metric representing the total energetic contribution of parents to their offspring (after Winemiller 1989) | PC |
| Total Fecundity | Total number of eggs or offspring per breeding season | TFEC |
| Egg size | Mean diameter of mature (fully yolked) ovarian oocytes (mm) | EGGS |
| **Movement classification** | No known movement associated with reproduction | MVT1 |
|  | Potamodromous (spawning migration) | MVT2 |
|  | Amphidromous | MVT5 |
|  | Anadromous | MVT6 |
|  | Catadromous | MVT7 |
| **Trophic guild** | Herbivore-detritivore (ca. > 25% plant matter) | TG1 |
|  | Omnivore (ca. 5 - 25% plant matter) | TG2 |
|  | Invertivore | TG3 |
|  | Invertivore-piscivore (>10% Fish) | TG4 |
| **Physiology** | Maximum temperature between cells occupied by species | TMAX |
|  | Minimum temperature between the cells occupied by species | TMIN |

| **References for more information on Methods:** |
| --- |
| Olden, J.D. & Kennard, M.J. (2010). Intercontinental convergence of fish life history strategies along a gradient of hydrologic variability. Pp. 83–107 In: Community Ecology of Stream Fishes: Concepts, Approaches, and Techniques. (Eds) K.B. Gido & D.A. Jackson. American Fisheries Society, Symposium 73, Bethesda, Maryland. |
|  |
| Sternberg, D. & Kennard, M.J. (2013). Environmental, spatial and phylogenetic determinants of fish life-history traits and functional composition of Australian rivers. Freshwater Biology. 58: 1767–1778. |
|  |
| Sternberg, D. & Kennard, M.J. (2014). Phylogenetic effects on functional traits and life history strategies of Australian freshwater fish. Ecography. 37: 54–64. |
|  |
| Sternberg, D., Kennard, M.J. & Balcombe, S.R. (2014). Biogeographic determinants of Australian freshwater fish life-history indices assessed within a spatio-phylogenetic framework. Global Ecology and Biogeography. 23: 1387–1397 |


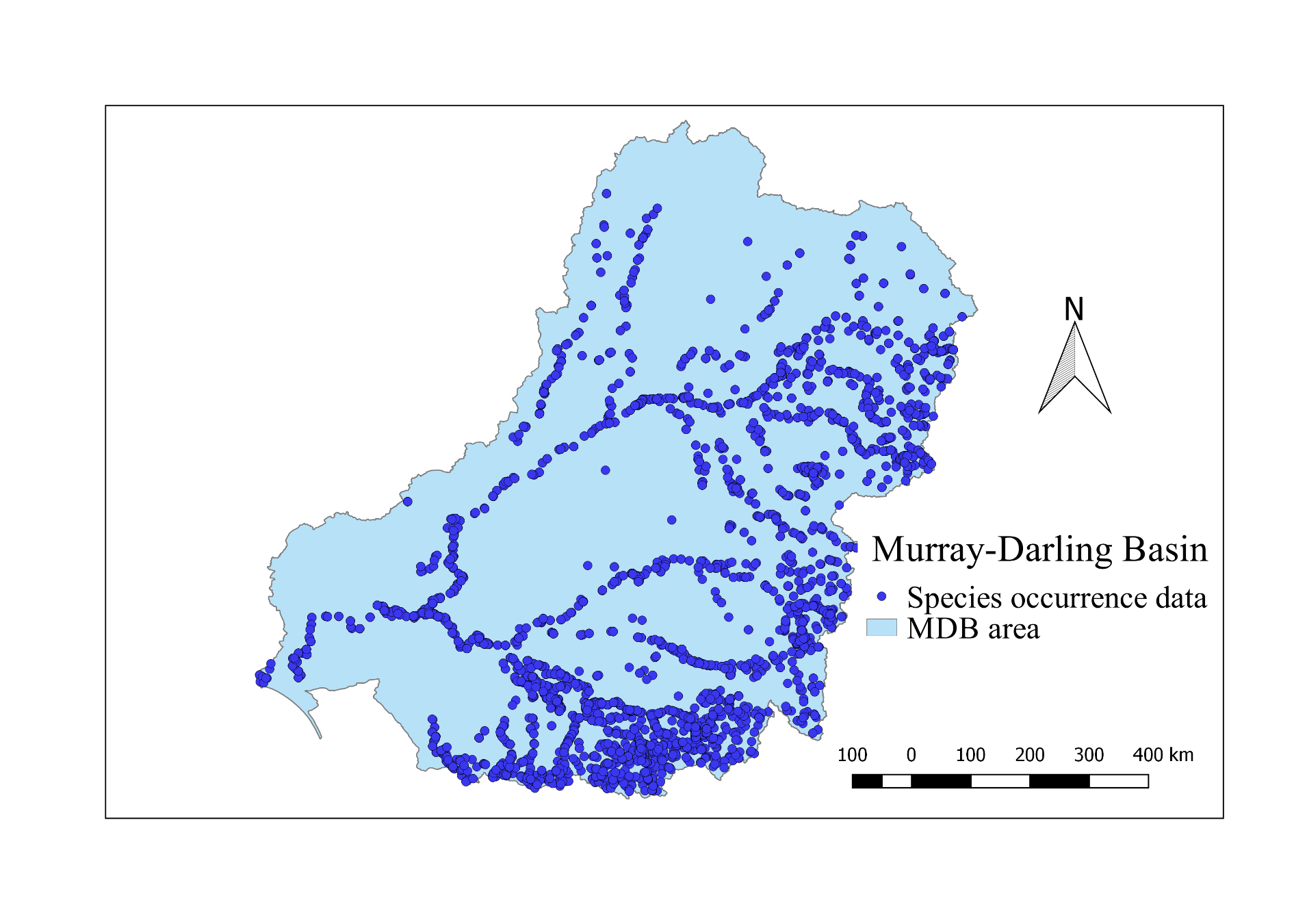


Fig A. Species occurrence data in the Murray-Darling Basin, Australia.

Table D. Indicator values for species’ functional traits in the different scenarios. Higher values represent a high proportional trait representation (i.e. fidelity) in a given scenario.

| **Trait** | **Present** | **2050 (4.5)** | **2050 (8.5)** | **2080 (4.5)** | **2080 (8.5)** |
| --- | --- | --- | --- | --- | --- |
| TMAX | 0.25131 | 0.12431 | 0.08541 | 0.08792 | 0.01668 |
| TMIN | 0.09725 | 0.08312 | 0.06963 | 0.07011 | 0.00652 |
| MAXL | 0.26315 | 0.12591 | 0.08426 | 0.08713 | 0.01497 |
| VPBEN | 0.25011 | 0.10723 | 0.06444 | 0.06705 | 0.01493 |
| VPNBEN | 0.23672 | 0.06032 | 0.04573 | 0.04943 | 0.00823 |
| LONG | 0.26485 | 0.12482 | 0.08227 | 0.08561 | 0.01615 |
| AGEMAT | 0.26206 | 0.12293 | 0.08455 | 0.08534 | 0.01642 |
| NONMOV | 0.22526 | 0.10311 | 0.06537 | 0.06991 | 0.01584 |
| POTAMO | 0.24022 | 0.09193 | 0.05151 | 0.05396 | 0.00598 |
| AMPHID | 0.01987 | 0.00341 | 0.00463 | 0.00388 | 0.00032 |
| CATAD | 0.06314 | 0.00003 | 0.00000 | 0.00001 | 0.00000 |
| PARC | 0.26145 | 0.10348 | 0.07318 | 0.07448 | 0.01606 |
| TFEC | 0.27991 | 0.12963 | 0.08141 | 0.08120 | 0.01416 |
| EGGS | 0.26379 | 0.12263 | 0.08420 | 0.08551 | 0.01628 |
| HERB-DET | 0.10373 | 0.07075 | 0.03001 | 0.03098 | 0.00030 |
| OMNI | 0.18755 | 0.05653 | 0.02389 | 0.02747 | 0.00222 |
| INV | 0.24535 | 0.09004 | 0.07251 | 0.07184 | 0.01624 |
| INV-PISC | 0.18063 | 0.05030 | 0.02849 | 0.01879 | 0.00718 |
